# An Energy-Efficient Mobile Sink-Based Unequal Clustering Mechanism for WSNs

**DOI:** 10.3390/s17081858

**Published:** 2017-08-11

**Authors:** Niayesh Gharaei, Kamalrulnizam Abu Bakar, Siti Zaiton Mohd Hashim, Ali Hosseingholi Pourasl, Mohammad Siraj, Tasneem Darwish

**Affiliations:** 1 Faculty of Computing, Universiti Teknologi Malaysia (UTM), 81310 Skudai, Johor, Malaysia; knizam@utm.my (K.A.B.); sitizaiton@utm.my (S.Z.M.H.); sjdtasneem2@live.utm.my (T.D.); 2 Faculty of Electrical Engineering, Universiti Teknologi Malaysia (UTM), 81310 Skudai, Johor, Malaysia; poorasl.ali@gmail.com; 3 College of Engineering, King Saud University, Post Box 800, Riyadh 11421, Saudi Arabia; siraj@ksu.edu.sa

**Keywords:** wireless sensor networks, network lifetime, energy holes, genetic algorithm, mobile sink

## Abstract

Network lifetime and energy efficiency are crucial performance metrics used to evaluate wireless sensor networks (WSNs). Decreasing and balancing the energy consumption of nodes can be employed to increase network lifetime. In cluster-based WSNs, one objective of applying clustering is to decrease the energy consumption of the network. In fact, the clustering technique will be considered effective if the energy consumed by sensor nodes decreases after applying clustering, however, this aim will not be achieved if the cluster size is not properly chosen. Therefore, in this paper, the energy consumption of nodes, before clustering, is considered to determine the optimal cluster size. A two-stage Genetic Algorithm (GA) is employed to determine the optimal interval of cluster size and derive the exact value from the interval. Furthermore, the energy hole is an inherent problem which leads to a remarkable decrease in the network’s lifespan. This problem stems from the asynchronous energy depletion of nodes located in different layers of the network. For this reason, we propose Circular Motion of Mobile-Sink with Varied Velocity Algorithm (CM2SV2) to balance the energy consumption ratio of cluster heads (CH). According to the results, these strategies could largely increase the network’s lifetime by decreasing the energy consumption of sensors and balancing the energy consumption among CHs.

## 1. Introduction

A Wireless Sensor Network (WSN) is comprehensively defined as a set of sensors located in an area to sense the environment. Sensors are able to gather different types of data based on their defined functions. After some processing operations, the collected data is sent to a base station (BS) or sink. This process is done by different routing methods. One of the components of the sensor is a battery or power source that is often limited. In addition, these sensors are usually located in areas inaccessible to humans; thus, it is not possible to recharge or exchange these components. For this reason, the power supplies of sensors should be taken advantage of to the fullest possible extent, this process is known as energy efficiency which can increase the network lifetime. Energy efficiency can be achieved by decreasing or balancing the energy consumption of nodes. Clustering is one solution to achieving energy efficiency. There are several reasons to apply clustering techniques in WSNs such as increased scalability, less load, less energy consumption, latency reduction, collision avoidance, guarantee of connectivity, fault tolerance, load balancing, energy hole avoidance and increasing network lifetime [1]. In addition, dividing the network area into subareas helps control the coverage hole problem [2], which appears whenever some parts of the network areas are not covered by any sensor nodes.

One important parameter in hierarchical routing protocols is cluster size. With small size clusters, networks may encounter connectivity and coverage problems. Furthermore, most existing clustering based algorithms have not been considered the network coverage to improve the network lifetime [3]. In [3], it is shown that balancing energy consumption per unit area can improve energy balancing ratio throughout the network and a Balanced Energy-Efficiency clustering algorithm (BEE) is proposed to extend coverage sensitive longevity. In [4], the authors showed that if the cluster size is not properly chosen, the total energy consumption of the network will increase exponentially, either when the cluster size is smaller than the optimal value or when the cluster size is larger than the optimal size. In addition, if this parameter is not chosen properly, decreasing the energy usage of member nodes, which is the one objective of applying clustering techniques, will not be achieved; hence, the clustering process will act contrary in this regard. Therefore, the energy consumption of nodes before clustering should be considered to determine the cluster size. Unfortunately, this rule was not considered in previous works. At any rate, grouping nodes into optimal clusters is an NP-hard problem [5] and therefore using optimization algorithms can be an effective method in this regard.

Moreover, in multi-hop clustering WSNs, CHs located around the BS relay the data packets from outer CHs to the sink. Accordingly, those located nearby the sink deplete their energy quicker than others. In this situation, the whole network will be partitioned while plenty of energy is left unused and energy holes appear, which can decrease the network’s lifetime [6]. In fact, this problem is one of the main causes of premature network death. Lian et al. [7] showed that up to 90% of the total energy of the network can be wasted when the entire network is subject to premature death. The techniques proposed to solve the energy hole problem are grouped into nodes distributions strategies [8,9,10], adjusting the data transmission range of nodes [11,12], usage of sink mobility [13,14], and adding relay nodes [15]. Furthermore, by optimizing the clustering technique parameters, the problem of energy holes can be mitigated. Parameters that impact the network lifetime include cluster size [16], the number of clusters, and the CH selection technique [6]. Recently, mobile agents such as mobile sinks, relay nodes and data collectors have been widely used to achieve energy efficiency [17]. One important parameter in mobility-based WSNs is the velocity of mobile sinks. Recently, this parameter has been adjusted to reduce end-to-end packet delays [18] and to increase the packet delivery ratio. Adjusting the velocity of MSs in different layers of the network can solve the energy hole problem, which was previously not considered.

The proposed algorithm in this paper is efficiently designed for circularly-symmetric WSNs since it is easy to abstract in a view of routing optimization and the energy consumption o can be easily controlled in this type of network [19]. In the first part of the proposed mechanism, the optimal cluster size interval is obtained. The lower bound interval is obtained according to the node density needed to achieve a coverage guarantee and the maximum value is calculated according to the energy consumption of sensor nodes before and after clustering to achieve energy efficiency. We use the two-stage Genetic Algorithm (GA) to find the upper bound interval and also to derive the exact value of the angle from the obtained interval. Thus, a coverage-guarantee and energy-efficiency-based unequal clustering technique is proposed is the first part of this paper. This is followed by the use of some mobile sinks to solve the energy hole problem. In the proposed method the velocity of mobile sinks is adjusted in different zones to balance the energy consumption ratio of CHs belonging to the different layers of the network. The remaining part of this paper is organized as follows: Section 1 develops the introduction. Section 2 provides a literature review of the research area. The system model is explained in Section 3. A mobility-based energy efficiency algorithm is introduced in Section 4. Numerical results and conclusions are finally given in Section 5 and Section 6, respectively.

## 2. Related Works

As mentioned in the previous section, grouping the nodes into optimal clusters is known as an NP-hard problem [5]. Consequently, one effective method for optimizing the cluster size is to use optimization algorithms. Latif et al. [20] used Particle Swarm Optimization (PSO) in their proposed protocol in order to determine the optimal cluster size by minimizing the distance between member nodes and CHs and decreasing the energy consumption of the network. Hussain [21] used GA to create optimal clusters for energy efficiency in WSNs. Elhoseny [22] proposed a self-clustering method for heterogeneous WSNs using a GA that optimized the network lifetime. In addition, there are other methods which adjust cluster size with different objectives. The designers of the LEACH protocol [23] have obtained an efficient cluster size so that the remarkable amount of energy can be saved. However, their results only determine an interval to which the optimal cluster size belongs. It can be shown that in many network configurations, the analytical results give a long interval and therefore, the optimal number of clusters must be found through simulations for all the numbers that belonged to the aforesaid interval [24]. An unequal cluster size (UCS) was proposed by Soro and Heinzelman [25] in order to balance the energy expenditure among CHs. UCS is the first unequal clustering model in a wireless sensor network to increase network life span, however, this model is not applicable to a larger scale WSN [1]. In [24] a mathematical framework is provided in order to obtain the optimal cluster size which can improve the network lifespan by decreasing the total energy expenditure of the network. The Distance-based Segmentation (DBS) protocol [26] provides a parallel version of the LEACH algorithm to solve the energy imbalance problem which occurs in LEACH. DBS is a cluster-based protocol that divides the network into equal area rings or coronas and applies different clustering rules to each segment to reduce the energy consumption and balance the energy consumption among sensors. In [27], the authors showed that the CH election manner can affect the size of clusters. Therefore, they tried to find the optimal number of clusters by choosing optimal CHs. Lai et al. in [28] attempted to balance the energy consumption of CHs by assigning the larger cluster sizes to CHs that have to forward fewer data in comparison with others. In [29], the authors proposed a cluster-based cooperative spectrum sensing algorithm to save energy consumption. CHs are selected based on a sensor’s location with respect to a fusion center (FC), its residual energy, and its signal-to-noise ratio (SNR). An adaptive fuzzy clustering protocol (called LEACH-SF) is proposed in [30] to achieve energy efficiency. They used a fuzzy c-means algorithm to solve the unbalanced clusters problem, and then CHs are elected based on the residual energy of nodes, the distance from the BS, and the distance from the cluster centroid. They used the artificial bee colony algorithm to adjust the fuzzy rules of their proposed protocol. The objective function of the algorithm is defined to enhance the network lifetime, based on the application specifications.

In [31] the authors applied a clustering technique on a corona-based WSN. In their model, the cluster size varies with the ring index and the size of clusters is equal in each ring. Their goal is to improve the network lifetime by achieving an energy balance among CHs and decreasing the total energy consumption of the network. However, in their model, the size of clusters is small and this property contrary to allowing the scalability of the network [32]. One goal of clustering is scalability of network management, however, if the cluster size is too small, this goal will not be achieved. On the other hand, if the size of clusters is too large, the intra-cluster energy consumption will increase. Moon et al. in [32] introduced an approach to alleviate the energy hole problem in a sink-centric traffic pattern network. In their model, each layer of the network is divided into equal clusters so that CHs are responsible for forming the clusters in cluster rings. In the uniform node distribution, the innermost CHs can preserve some energy for the inter-cluster traffic, if clusters belonging to the innermost layer have a smaller size than outer layers. However, this principle is not fulfilled in the method proposed in [32]. In addition, one goal of applying clustering is to decrease the energy consumption of nodes. However, this aim will not be realized if the cluster size value is not properly chosen. In fact, the clustering technique will only be effective if the consumed energy ratio by nodes is decreased after applying clustering. Therefore, the energy consumption of nodes before clustering should be considered to determine the cluster size. However, this rule was not considered in previous works.

Furthermore, there are several strategies proposed in order to mitigate the energy hole problem. The authors of [12] proposed the Energy Balancing Cluster Head (EBCH) method which is based on adjusting the data transmission range of nodes. In their model, CHs forward the aggregated data packets after bisecting them, so that data packets will be sent to the base station by two different data transmission manners; single- and multi-hop. In [33] the Archimedes spiral node deployment strategy is proposed to optimize the network lifespan. For this reason, the authors introduced a routing aware clustering strategy to balancing the energy expenditure among CHs. However, non-uniform node deployment strategies are not practical for networks which are inaccessible to humans. Among the proposed methods, sink mobility is an important technique in improving network lifespan and energy efficiency [34]. Using mobile sinks can reduce the burden of energy consumption from the sensors to sinks, which are typically considered to have unlimited energy supply and larger computational power. In addition, this method has been accepted as an effective technique for mitigating the energy hole problem by avoiding extreme transmission overhead at the nodes located around the base station. Generally, using mobile sinks in the network offers many benefits such as mitigating the energy holes, increasing the network lifetime, reducing the drop packet ratio, decreasing the energy consumption, increasing the security and providing connectivity in the network [35]. With regards to the advantages of using mobile sinks, the outcome of the network can depend on type of sinks are used. The basic idea of mobile sinks was proposed by Shah et al. [36] where mobile sinks are called “data mules”. In their work, the mules perform a random walk in their close vicinity to aggregate the data packets and then they drop off the data at some access points. Since the transmission range of nodes is short, their energy consumption can be greatly reduced. Wang et al. [37] proposed a Mobility-Based Data Collection Algorithm in order to improve the network lifetime. In their strategy, the area of the network has a circular shape with MSs working in a back-and-forth motion in the periphery of the circle. In [38], Wang et al. introduced a mobility-based technique in order to optimize the clustering algorithm and sink node deployment strategy under the smart home network concept. They considered two different conditions with a different number of mobile sinks. First, they used a single sink which has a circular motion in a different radius. Then, they used multi mobile sinks to find the optimal number of mobile sinks in a circular area. They considered that the mobile sinks moved with constant velocity. One important parameter of mobility-based strategies is the velocity of the mobile agent. In [39], the velocity of the mobile sink is reduced to guarantee message delivery. This scheme could enhance the network lifetime and achieve a high packet delivery ratio by using multiple mobile sinks with a fixed speed. However, the reduced sink velocity increased the data delivery latency [40], whereas, using both types of sink can solve the data delivery latency problem and also increases the packet delivery ratio. This paper attempts to solve the energy hole problem by engaging the MSs with unequal velocities. In addition, it is considered that the velocities of MSs are separately adjusted in different angles.

## 3. System Model

### 3.1. Node Properties

In this work, it is considered that sensor nodes and sinks have the following properties:

1. There is one static sink located in the center of the area with unlimited energy supply.
2. Sensor nodes cannot move after they are deployed.
3. Sensor nodes are powered by limited energy supplies.
4. Transmission range of sensor nodes is adjusted after determining the size of clusters and based on the distance between nodes and their CHs.
5. The network is homogenous. All sensors have similar properties, such as data transmission range, initial energy, etc.
6. Multi hop data transmission model is applied for inter cluster communication.
7. Member nodes transmit their data packets directly to their local CHs.
8. The number of mobile sinks is equal to the number of coronas.
9. Mobile sinks are resource-rich devices.
10. Mobile Sinks and static sink are able to communicate with each other.
11. Static sink determines and sets the velocities of mobile sinks.

### 3.2. Network Area

**Figure 1:**
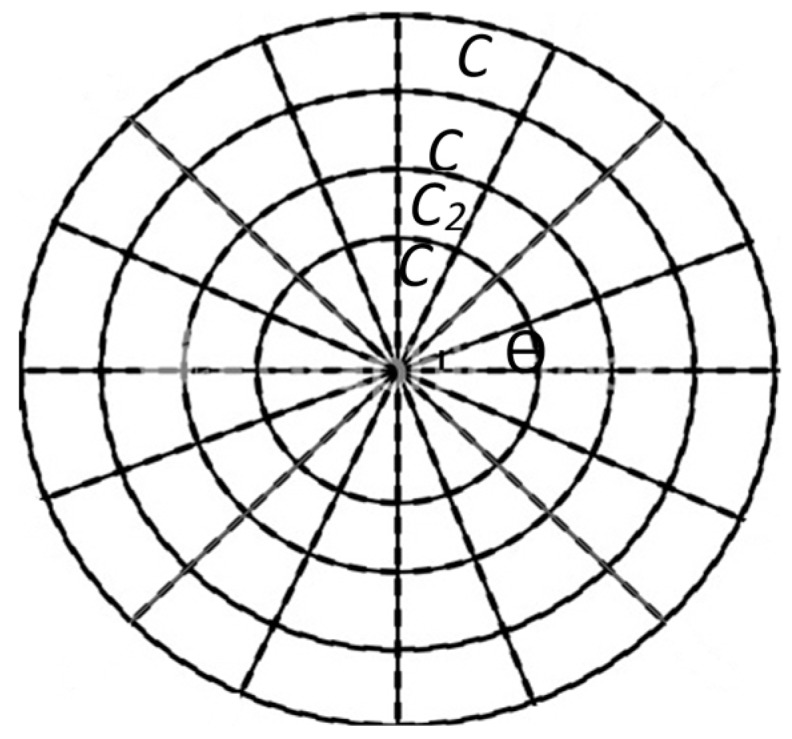
Clustered corona-based WSN (C is the corona number and ϴ denotes the angle of each sector).

We assume that all sensor nodes are uniformly distributed throughout the network. It is considered that the area of the network is a circle with radius R. The network is divided into K adjacent coronas, rings or annuli. This division must guarantee the maximum energy equilibrium [41]. The sensors are deployed throughout these zones and the sink is located at the center of this area and coronas have the same width (r). Networks with these properties are called simple corona-based WSNs (SCWSNs) [15,19]. However, in our model the coronas are divided into sectors acting as clusters with a constant angle (ϴ) as shown in Figure 1 which is called a clustered corona-based WSN (CCWSN) [12,25,34,42]. By applying the clustering routing technique in SCWSNs, network scalability and easier management will be guaranteed [38]. The nodes in each cluster send their data packets toward the local CHs. In each round, every CH receives and aggregates the packets from local member nodes and also from outer CHs located in the same sectors. Data packets aggregated by CHs will be sent to their inner CHs after compressing by a factor β, similar to the model proposed in [25].Suppose this is a uniform deployment with ‘ρ’ density, all sensor nodes have the same initial energy (ɛ0) and generate l bit data packet per second.

### 3.3. Energy Model

We use the radio model applied by Soro and Heinzelman [25] where the transmitter divides the energy to run the radio electronics and power amplifier and the receiver also divides the energy only to run the radio electronics. In this model the energy usage for transmitting and receiving l bit of data will be as follows:

(1) $$E_{tx}=l\left(E_{elec}+\propto d^{n}\right)$$

(2) $$E_{rx}=l\left(E_{elec}\right)$$

where *l* is the length of the transmitted/received message in bits. ∝ denotes the energy dissipated by the op-amp in data transmission and n is the path loss exponent and depends on the specific propagation. For example, in free space, this value will be 2. *d* is the distance between the destination and source. Another parameter *Eelec* denotes the electronic energy depending on the digital coding, filtering and spreading of the signal. The parameters are listed in Table 1.

## 4. Proposed Algorithm

One purpose of using clustering techniques in WSNs is to decrease the energy consumption of sensor nodes. Accordingly, one objective of applying clustering on a simple corona-based WSN (SCWSN) is to reduce the energy consumption of nodes. Selecting the optimal cluster size can be effective in this regard [27]. In CCWSN, the cluster size depends on the angle of the sectors (*ϴ*). If this angle is too large, the distance between member nodes and local CHs will increase and energy consumption of member nodes will be more than their energy consumption in SCWSNs. Then, in this situation, clustering on corona-based WSN cause an increase in the energy usage of sensor nodes. On the other hand, if this angle is too small, the network may encounter connectivity and coverage problems. Therefore, an optimal cluster size interval is needed. In our model, the lower bound of this interval is calculated based on the lowest number of nodes in the interior zone according to the density of nodes. The upper bound is obtained based on the comparison between the total transmission range of nodes in the SCWSN and the total distance between member nodes and local CHs in CCWSN. We use the two-stage GA (GA) to obtain the maximum angle and also derive the exact value of the angle from this interval. At the first stage, we define a new cost function, with the objective of minimizing the difference between total transmission range of nodes in SCWSN and the total distance between member nodes and local CHs in CCWSN for obtaining the maximum value of the interval. Then, the exact value of the angle is derived from this interval at the second stage with the objective of minimizing the total energy consumption of network.

In the second part of this work, the Circular Motion of MSs with Varied Velocity (CMS2V2) algorithm is proposed to solve the unbalanced energy consumption of CHs in different coronas. For this reason, some MSs which have a circular motion in coronas are used to compensate the extra energy usage of CHs. In CMS2V2 algorithm the velocity of MS belonging to the *i*th corona (except for innermost corona) is regulated in the different angles (from (*j*-1)th sector to *j*th sector) in a way that the total energy consumption of CH belonging to *i*th corona and *j*th sector is balanced with the energy consumption of CH located in 1st corona and *j*th sector. In fact, by decreasing the velocity of MSs belonging to outer coronas, a chance is given to inner MSs to increase their sojourn time in clusters. The details of the CMS2V2 strategy are described in the algorithm flow of this mobile sink-based strategy (Algorithm 1).
**Algorithm 1**
*Circular Motion of MSs with Varied Velocity Algorithm*
Initialize all the parameters;Round = 0;Static sinks computes the velocities of MSs based on Equation (30);MSi Starts to move in predetermined circular path from position (((2*j*-1)(d/2)), 0)Turn Round = Turn Round +1;J = 1;**// sector number**Stops at (((2*j*-1)(d/2).cos(ϴ/2) + Xcenter), ((2*j*-1)(d/2).Sin(ϴ/2) + Xcenter)); **//MS stops at the bisector of sectors**MS sends a Hello packet to the nearest CH;CH sends a data packet to the MS **//data packet contains corona number, sector number and waiting time parameter;**MSi Computes the sojourn time in Cluster(i, j) based on Equation (30);MS propagates Hello packets; **// Hello packet contains of position of MS and cluster id**the MNs which are located in the cluster which is mentioned in received packet will connect to MS;Set timer = ST(i,j,Round);While timer > 0 doCH Temporarily stops its operations;MSi aggregates the data packets of nodes belonging Cluster (i, j);MSi receives the data packets from CH (i-1, j) and nodes belonging Cluster (i, j);Check timer;End whileJ = j + 1;MSi Computes its velocity {MSi | 1 < I ≤ k} based on Equation (30);MSi Starts to move;Check the current position;If current position = (((2*j*-1) (d/2)), 0); **// when the angle of position of MSs is 0.**Go to 5;End IfGo


### 4.1. Related Concepts

#### 4.1.1. Balancing the Energy Consumption Ratio

Based on the definition of network lifespan proposed by Soro and Heinzelman [25] the network lifespan is the time when the first CH exhausts its energy supply. Since the nodes are uniformly distributed in the network, the number of nodes in *i*th corona (C_i_) and the total number of nodes in the network can be obtained respectively as follows [43]:

(3) $$N_{i}=\left(2i-1\right)N_{1}$$

(4) $$M=\sum_{i=1}^{k}\left(2i-1\right)N_{1}=k^{2}N_{1}$$

where k is the number of coronas in the network. Since the nodes are uniformly distributed throughout the network, the density of nodes is equal in every part of the network as follows:

(5) $$\rho =\frac{M}{S}$$

ρ and S denote the density of nodes and the area of the network, respectively. If the area of the innermost corona is $S_{1}=\pi r^{2}$ which r denotes the width of coronas then the area of *i*th corona can be calculated via the area of the inner most corona [43]:

(6) $$S_{i}=S_{1}\left(2i-1\right),\;S_{1}<S_{2}<S_{3}<\ldots <S_{k}$$

The energy usage of CHs for aggregating, receiving and transmitting the data packets of local member nodes is measured as follows:

(7) $$ECHintra_{i}=l\left(\left(N_{i}-1\right)\left(E_{rx}\right)+\left(N_{i}\right)\left(E_{agg}\right)+\left(N_{i}\cdot \beta \right)\left(E_{tx}\right)\right)$$

β denotes the compression ratio and can be in the range [1Ni,1], that β=1Ni denotes the perfect aggregation and $\beta =N_{i}$ denotes the CH does not perform any aggregation [25]. We considered the perfect aggregation when every CHs compresses their received data packets from its cluster into one outgoing packet. In the multi-hop data transmission model, CHs located in *i*th corona have to relay the data packets from *j*th corona {C_j_ |i+1≤j≤k}. Thus, the energy consumption of CHi in CCWSN can be written as follows:

(8) $$ECH_{i}=l\left(\left(\left(N_{1}\left(k^{2}-\sum_{j=1}^{i}\left(2j-1\right)\right)\right)\cdot \beta \right)\left(E_{rx}\right)+\left(\left(N_{1}\left(k^{2}-\sum_{j=1}^{i}\left(2j-1\right)\right)\right)\cdot \beta \right)\left(E_{tx}\right)\right)+ECHintra_{i}$$

For CH election, we used the model proposed in [12]. In their model, a centroid region with radius ‘λ’ is considered in clusters. The distance between nodes and centroid points of clusters will be estimated. If the distance is less than the radius of centroid regions then these nodes will be recognized as centroid region nodes or CHs candidates. In each round, the nodes which have the maximum residual energy and minimum distance to centroid point will be elected as CH.

Since there is a monotonically increasing sequence relationship between the areas of adjacent clusters in the same sector and different coronas, the area of centroid region would increase similarly. Furthermore, this value will be obtained with respect to the area of innermost centroid region:

(9) $$Area_{i}=\left(2i-1\right)Area_{\;1}$$

Since the uniform node distribution has been applied, then the number of nodes in centroid region of clusters in innermost corona with respect of density of nodes will be calculated as follows:

(10) $$NCH_{1}=\rho \times Area_{\;1}$$

Thus, the number of centroid region nodes in the clusters of *i*th corona will be estimated as follows:

(11) $$NCH_{i}=\left(2i-1\right)NCH_{1}$$

#### 4.1.2. Load Balancing

In multi-hop clustering WSNs, the CHs located around BS have to relay not only the data packets belonging to own member nodes, but also they have to receive and transmit the data packets of outer clusters. Thus, the innermost CHs with maximum energy consumption ratio are critical, however, the CHs of outermost clusters have a minimum energy consumption ratio in comparison to others. Our main reason is to balance the energy consumption ratio of CHs located in different coronas in CCWSN. Energy consumption ratio is defined as the energy usage of CHs to the number of nodes located in the centroid region as CH candidates [44]. By equalizing the energy consumption ratio of CHs belonging to *i*th corona with innermost corona, we have:

(12) $$\frac{ECH_{1}}{NCH_{1}}=\frac{ECH_{i}}{NCH_{i}}$$

By substituting the Equation (11) in the equation above, the following equation will be derived:

(13) $$ECH_{i}=\left(2i-1\right)ECH_{1}$$

From Equation (13), it is concluded that to balance the energy consumption ratio among coronas, the CHs located in the innermost corona should consume (2i−1) times less energy than the CHs belonging to the *i*th corona.

### 4.2. Cluster Size Optimization in Clustered Corona Based WSNs

In this section the optimal interval of the angle will be formulated.

#### 4.2.1. Minimum Angle of Sectors

The lower bound of the cluster size interval is obtained according to the density of nodes in the network. If the cluster size is too small, some interior clusters will be empty; thus, networks encounter connectivity and coverage problems. In a corona based WSN, according to Equation (3), a corona will be empty if, and only if, the innermost corona is empty:

(14) $$N_{i}=0\;IIFN_{1}=0,\;2\leq i\leq k$$

**Figure 2:**
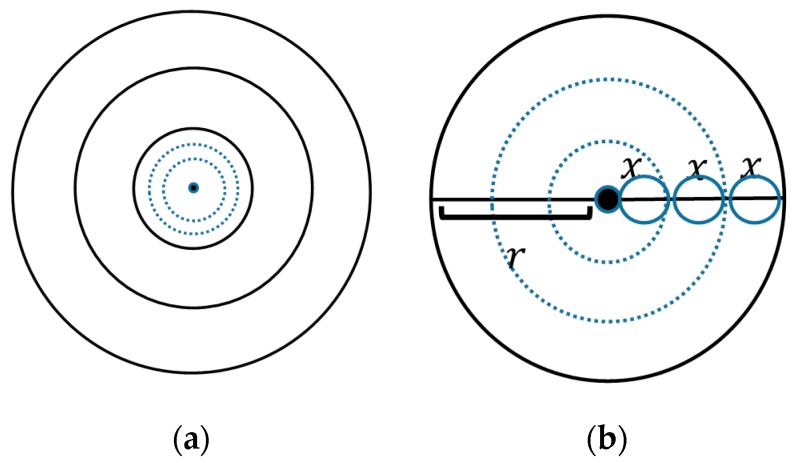
(**a**) Dividing the innermost corona into sub-coronas; (**b**) enlarged view of the innermost corona.

To calculate the lower bound of the interval, it is considered that interior zones of the clusters belonging to innermost corona have the lowest number of nodes. With respect to the uniform distribution of nodes, the allocated space to nodes will be as follows:

(15) $$space=\frac{S}{M}$$

where *S* and *M* denote the area of the network and the total number of nodes in the network, respectively. The innermost corona is divided into sub-coronas with equal thickness as shown in Figure 2.

There is a relationship between the number of nodes, sectors and sub-coronas in innermost corona, which can be written as follows:

(16) $$a\cdot \sum_{j=1}^{b}\left(2j-1\right)=N_{1}\;or\;a\times b^{2}=N_{1}$$

where a denotes the number of sectors with the minimum angle which should be obtained in this section, b shows the number of sub-coronas of the innermost corona. $N_{1}$ is the number of nodes located in the innermost corona. In order to calculate the number of sub-coronas in the innermost corona, the width of sub-coronas should be obtained. It is considered that the occupied space by nodes is circular, then, the diameter of the occupied space demonstrates the thickness of each sub-corona which can be given by:

(17) $$x=\sqrt{\frac{space}{\pi }}\times 2$$

By dividing the width of the coronas by the width of sub-coronas the number of sub-coronas can be obtained as follows:

(18) $$b=\lfloor \frac{r}{x}\rfloor$$

By substituting Equation (18) in Equation (16) the number of sectors is derived as:

(19) $$a=\frac{N_{1}}{b^{2}}$$

It is considered that each sector has only one sensor node in the innermost sub-corona, so the minimum angle of sectors is as follows:

(20) $${ϴ}_{Min}=\frac{360}{a}$$

#### 4.2.2. Determining the Maximum Angle and Exact Angle from the Interval by Two-Stage GA

##### First Stage (Maximum Angle)

The maximum value of the interval is estimated at the first stage of the proposed GA. In our strategy, solutions are represented in binary as strings of 0s and 1s. The sum of the decision of variables in each chromosome denotes the number of sectors. Since the energy consumption of nodes depends on their data transmission range, the objective is to minimize the differences between the total transmission range of nodes in SCWSN and the total distance between member nodes and local CHs in CCWSN as follows:

(21) $$f\left(x\right)=(\left(M\cdot r\right)-\sum_{s=1}^{k}\sum_{i=1}^{x}\sum_{j=1}^{\frac{N_{s}}{x}}d_{toCH}\left(j\right){)}^{2}\;\text{Subject to}:\;{ϴ}_{Min}<x$$

where M denotes the number of node in the network and r denotes the thickness of coronas or the data transmission range of sensor nodes in SCWSN. *d_toCH_* is the distance between sensor nodes and local CH and Ni is the number of nodes in *i*th corona. *x* is the number of sectors which is generated by the GA.

##### Second Stage (Exact Angle)

In the second stage of the two-stage GA, the optimal angle is derived from [Min,Max] where min and max denote the lower and upper bound of variables, respectively. The objective is to minimize the total energy consumption of clusters:

(22) $$g\left(y\right)=\left(\sum_{s=1}^{k}\sum_{i=1}^{y}\left(E_{CH}\left(s,i\right)+\sum_{j=1}^{\frac{N_{s}}{y}}E_{MN}\left(j\right)\right)\right)\;\text{Subject to}:\;{ϴ}_{Min}\leq y\leq f\left(x\right)$$

where y is a randomly generated number between [Min , Max] which denotes the number of sectors. $E_{MNi}$ and $E_{CH}$ denote the energy usage of member nodes and CHs, respectively.

### 4.3. Circular Motion of MSs with Varied Velocity Algorithm (CMS2V2)

**Figure 3:**
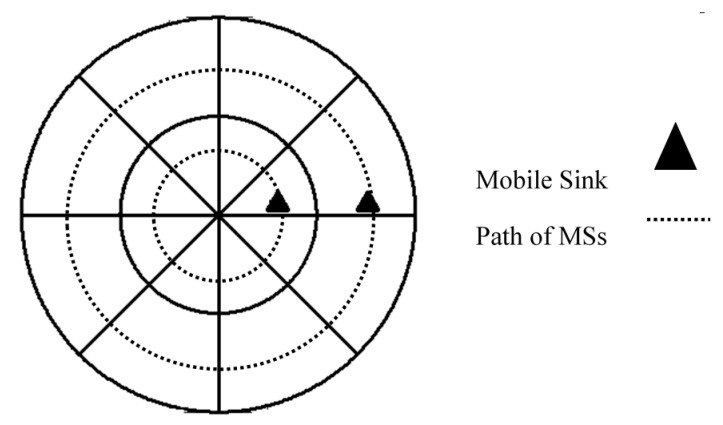
Circular motion of MSs in CCWSN**.**

We use MSs to balance the energy consumption among CHs in different coronas. The velocity of mobile agents is an important parameter in mobile sink-based WSNs. Adjusting this parameter in different locations can solve unbalanced energy consumption of CHs in different layers of the network. In CMS2V2 the MS belonging to the *i*th corona {Ci |1≤i≤k} identified by its unique id {i |1≤i≤k}. MSs have circular motion in their coronas as shown in Figure 3 and their velocities vary in the different angles to achieve a balanced energy consumption ratio among CHs located in different layers. The MS belonging to the critical corona (MS1) moves with constant speed. However, the velocity of MS belonging to *i*th corona {i |1≤i≤k−1} is regulated by static sink in the different angles (from (*j*-1)th sector to *j*th sector) in a way that the total energy consumption of CH belonging to *i*th corona and *j*th sector is balanced with the energy consumption of CH located in 1st corona and *j*th sector. In fact, by decreasing the velocity of MSs belonging to outer coronas, a chance is given to inner MSs to increase their sojourn time in clusters and compensate the extra energy consumption of CHs, thus:

VelocityMS_1_ > VelocityMS_2_ > VelocityMS_3_ > … > VelocityMS_k_ (23)

The velocity of *i*th MS, between two angles is shown as Velocity (Sid, SrartAngle, StopAngle, Round), where Sid denotes the id of MS and SrartAngle and StopAngle denote the moving angle interval of MS in Roundth turn number of MS. Here, the term Round means a cycle of rotating MSs and returning to the starting point. Since MSs are supposed to sojourn at the bisector of the angle of sectors (*ϴ*), Start and stop angle between (*j*-1)th and *j*th sectors are defined as follows:

(24) $$StartAngle=\left(2\left(j-1\right)-1\right)\frac{ϴ}{2}$$

(25) $$StopAngle=\left(2j-1\right)\frac{ϴ}{2}$$

In the proposed strategy, whenever a MS arrived at the bisector of angle (*ϴ*) in each sector it remained in the cluster for a limited time to serve the member nodes. In fact, the sojourn time is the time that the mobile sink spends at clusters to collect data from the sensor nodes and during this limited time CHs act as ordinary nodes in clusters. This is denoted as ST(i,j,Round); i and j denote the corona and sector number of cluster served by MS. CHs have to wait for MS, this waiting time parameter captures the total time spent by the MS in previous clusters during current round and in next clusters during previous round and also path length passed by MS with different velocities. This waiting time can be written as follows:

(26) $$WT\left(i,j,Round\right)=PL_{i}\;+\;\sum_{n=1}^{j-1}ST\left(i,n,Round\right)+\sum_{n=j+1}^{last\;sector}ST\left(i,n,Round-1\right)$$

where ST Shows the sojourn time of *i*th MS in other clusters. $PL_{i}$ denotes the time spent by MS belonging to *i*th corona for when moving along a path. Since $PL_{i}$ depends on the velocity of MS in different sectors, it can be written as:

(27) $$\begin{array}{l} PL_{i}=\sum_{n=1}^{j-1}\frac{ϴ\cdot \left(2i-1\right)\left(\frac{r}{2}\right)}{Velocity\left(i,\left(2n-1\right)\left(\frac{ϴ}{2}\right),\left(2\left(n+1\right)-1\right)\left(\frac{ϴ}{2}\right),Round\right)}\\ \;+\;\sum_{n=j+1}^{last\;sector}\frac{ϴ\cdot \left(2i-1\right)\left(\frac{r}{2}\right)}{Velocity\left(i,\left(2n-1\right)\left(\frac{ϴ}{2}\right),\left(2\left(n+1\right)-1\right)\left(\frac{ϴ}{2}\right),Round-1\right)} \end{array}$$

In addition, PLi denotes the time spent by the MS1 when moving along a path at constant velocity, which can be written as follows:

(28) $$PL_{1}=\left(\frac{2\pi \cdot \left(\frac{r}{2}\right)}{velocity1}\right)$$

The energy consumption of CH (i,j) during this waiting time can be calculated by Equations (8) and (26) as follows:

(29) $$EU\left(i,j\right)=\frac{WT\left(i,j,Round\right)}{t}\cdot ECH_{i}$$

where *t* unit is the time required to send l data packet to BS/CH/MS per second. By substituting Equation (29) in Equation (13) to achieve the balanced energy consumption between CH(*i*,*j*) and, CH(1,*j*) the velocity of the MS*_i_* between the sector numbers j and (*j*-1) should be as follows:

(30) $$Velocity\left(i,\left(2\left(j-1\right)-1\right)\left(\frac{ϴ}{2}\right),\left(2j-1\right)\left(\frac{ϴ}{2}\right),Round\right)=\frac{\left(2k-1\right)\cdot \left(ϴ\cdot \left(2i-1\right)\left(\frac{d}{2}\right)\right)\cdot ECH\left(i\right)}{t\left(\left(2i-1\right)EU\left(1,j\right)-EU\left(i,j\right)\right)}$$

MS_i_ starts to move from the *j*th sector into its trajectory after ST time as long as the MS_k_ arrives at the outermost cluster in the same sector; therefore, the sojourn time of MSi (2≤i<k) in the cluster (i,j) can be written as follows:

(31) $$\begin{array}{cc} ST\left(i,j,Round\right)= & \frac{ϴ\cdot \left(2k-1\right)\left(\frac{d}{2}\right)}{Velocity\left(k,\left(2\left(j-1\right)-1\right)\left(\frac{ϴ}{2}\right),\left(2j-1\right)\left(\frac{ϴ}{2}\right),Round\right)}\\ & -\frac{ϴ\cdot \left(2i-1\right)\left(\frac{d}{2}\right)}{Velocity\left(i,\left(2\left(j-1\right)-1\right)\left(\frac{ϴ}{2}\right),\left(2j-1\right)\left(\frac{ϴ}{2}\right),Round\right)}+\delta \end{array}$$

where δ denotes the sojourn time of MS belonged to the last corona. In addition, the sojourn time of MS_1_ in cluster (1,j) can be written as:

(32) $$ST\left(1,j,Round\right)=\frac{ϴ\cdot \left(2k-1\right)\left(\frac{d}{2}\right)}{Velocity\left(k,\left(2\left(j-1\right)-1\right)\left(\frac{ϴ}{2}\right),\left(2j-1\right)\left(\frac{ϴ}{2}\right),Round\right)}-\frac{ϴ\cdot \left(\frac{d}{2}\right)}{velocity\;1}+\delta$$

## 5. Performance Evaluation

### 5.1.Basic Description of the Simulation

We used Matlab to evaluate our proposed method in this paper. The impact of the angle of sectors on energy consumption of network in CCWSN was evaluated. In addition, total energy consumption of our cluster size optimization method is compared with three other technique; EBCAG [31], DBS and corona based WSN without applying clustering. Then, the performance of CMS2V2 method was assessed in terms of residual energy of the network and energy consumption ratio. Moreover, the network lifespan in three methods, MMSR [37], LEACH, Random-Movement, and CMS2V2 were compared under the parameters listed in Table 2.

### 5.2. Cluster Size Optimization

**Figure 4:**
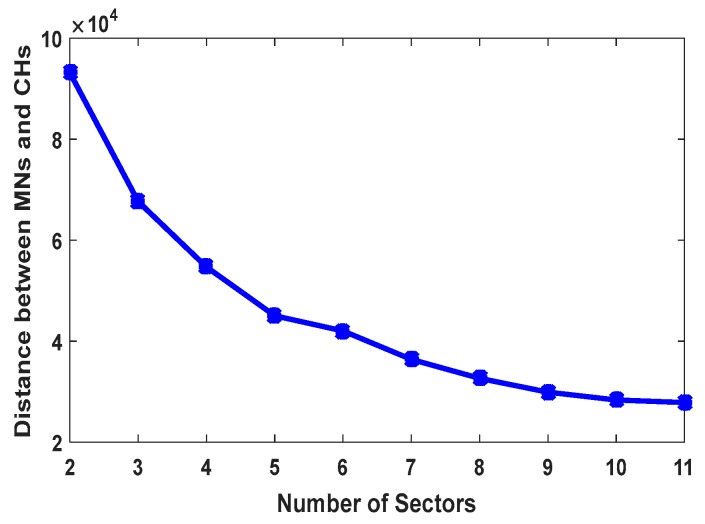
The influence of the angle of sectors on.

**Figure 5:**
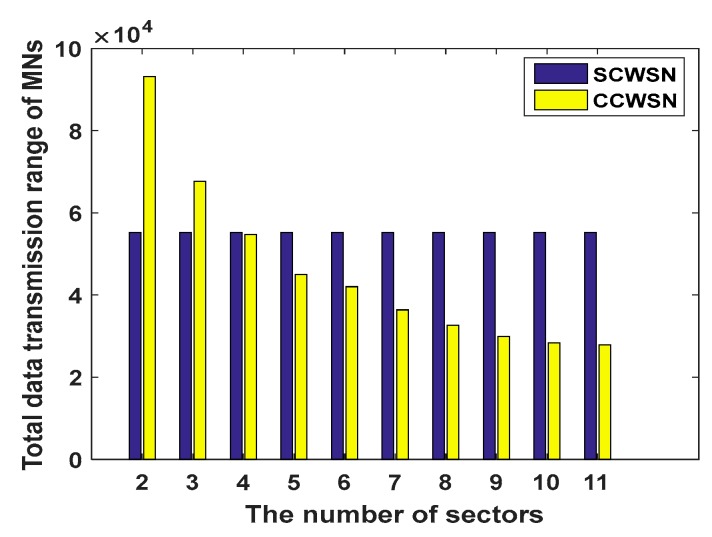
The comparison of data transmission range of MNs in CCWSN and SCWSN.

In the experiments, 300 nodes are uniformly distributed in an area with a radius of 1000 m^2^. All sensor nodes have same initial energy. Figure 4 shows the influence of different angles on the total transmission ranges of member nodes located in the last corona in CCWSN. Since in our model 32° is obtained as the minimum angle from Section 4.2.1, the angles which are less than this value are ignored. Figure 5 shows the comparison between SCWSN and CCWSN with different sector numbers in terms of total data transmission range of nodes. Based on Figure 5, when the network is divided into four sectors, there is the smallest difference between total data transmission range of nodes in SCWSN and CCWSN, thus 90° is considered as the maximum angle or upper bound of the interval.

**Figure 6:**
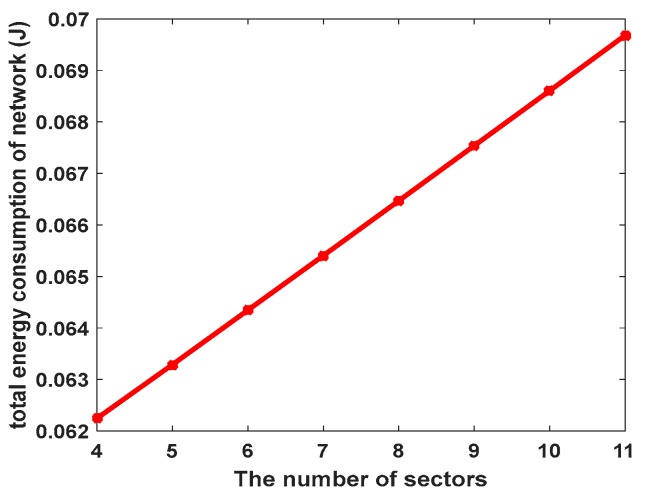
Total energy consumption of network in one round.

**Figure 7:**
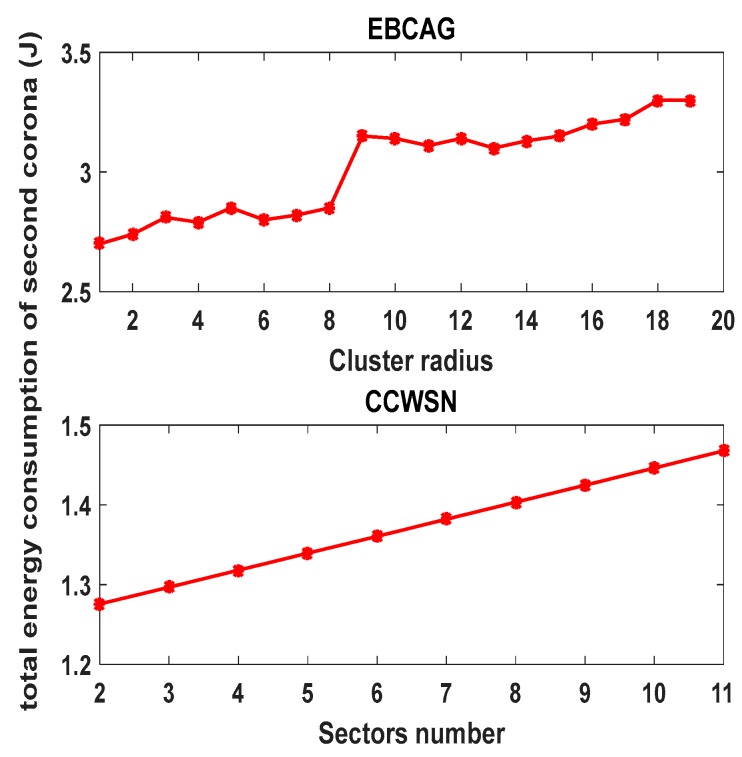
Total energy consumption of the second corona.

**Figure 8:**
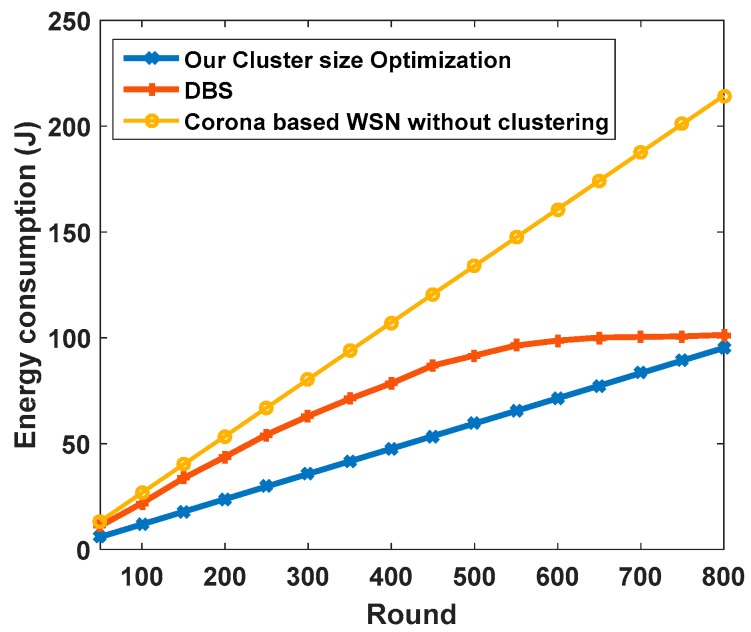
Total energy consumption in our proposed method, DBS and corona-based WSN without applying clustering.

Figure 6 shows the total energy consumption of network in one round. As can be seen, the total energy consumption of the network will rise by increasing the number of sectors. The number of sectors with the maximum and minimum angle and the number of sub-coronas of innermost corona have been calculated with the different number of nodes. Details of this evaluation are shown in Table 2. Since there is a direct relationship between increasing the cluster radius and the increase of the total energy consumption of the 2nd ring in EBCAG [31], this corona is the critical one in that method. Therefore, Figure 7 demonstrates the total energy consumption of only second corona in different cluster size in EBCAG [31] and CCWSN. As shown, EBCAG and CCWSN behave differently from each other by increasing the cluster size so that increasing the cluster radius in EBCAG leads to increase the energy consumption while it acts to the contrary in CCWSN. However, the energy consumption of 2nd corona in CCWSN model is lower than EBCAG and the remarkable amount of energy can be saved in our method. Moreover, Figure 8 compares the total energy consumption of network versus simulation time.

From Figure 8, it can be seen that remarkable amount of energy can be saved by applying the clustering on corona-based WSN. Furthermore, since our cluster size optimization method consumes less energy in comparison with DBS [26] strategy, CCWSN is close to the optimal solution.

### 5.3. Comparison of Network Lifespan

**Figure 9:**
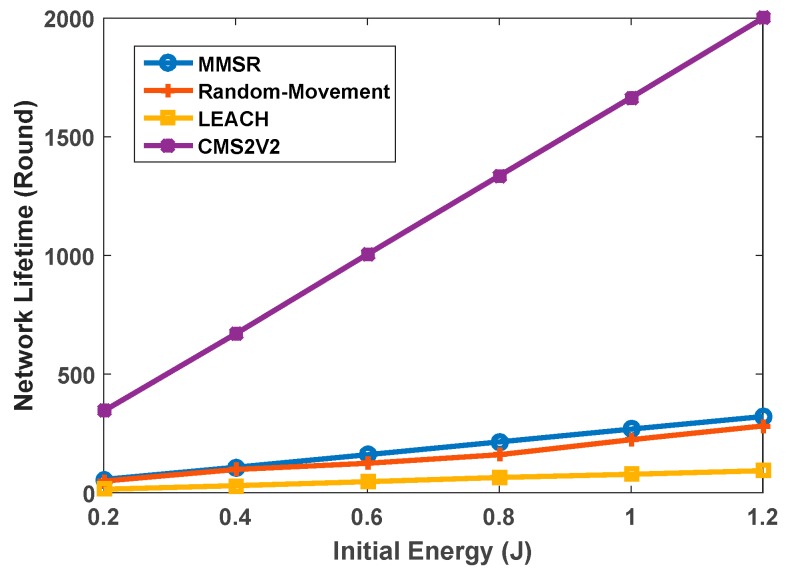
Network lifetime.

In order to analyze the network performance, we compare our proposed algorithm with three other different methods: MMSR [37] which uses a mobile sink to collect data packets and the Random-Movement technique where two MSs move randomly throughout the network and LEACH which uses one static sink. We define network lifespan as the time when the first CH exhausts its energy supply [25]. Figure 9 depicts the comparison of network lifespan of MMSR, Random-Movement, LEACH, and CMS2V2. As shown in Figure 9, despite the fact that MMSR and Random-Movement techniques use MSs, they do not bring a remarkable improvement in comparison with LEACH. As can be seen, the network lifespan in CMS2V2 is longer than in other strategies. This is because the CMS2V2 algorithm takes the waiting time of CHs into account to compensate for the extra energy consumption of CHs. The network lifespan in our model is six, seven and eight times more than the MMSR, Random-Movement, and LEACH, respectively. Related parameters are listed in Table 3.

**Figure 10:**
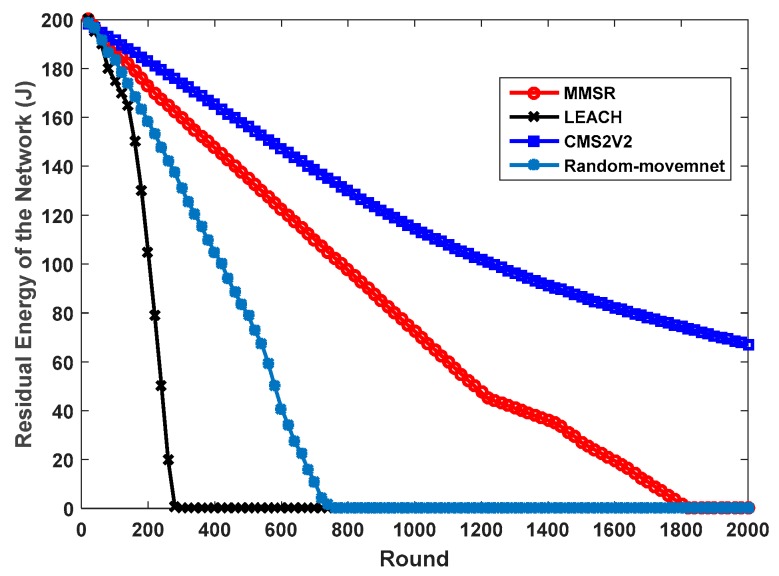
Residual energy comparison.

**Figure 11:**
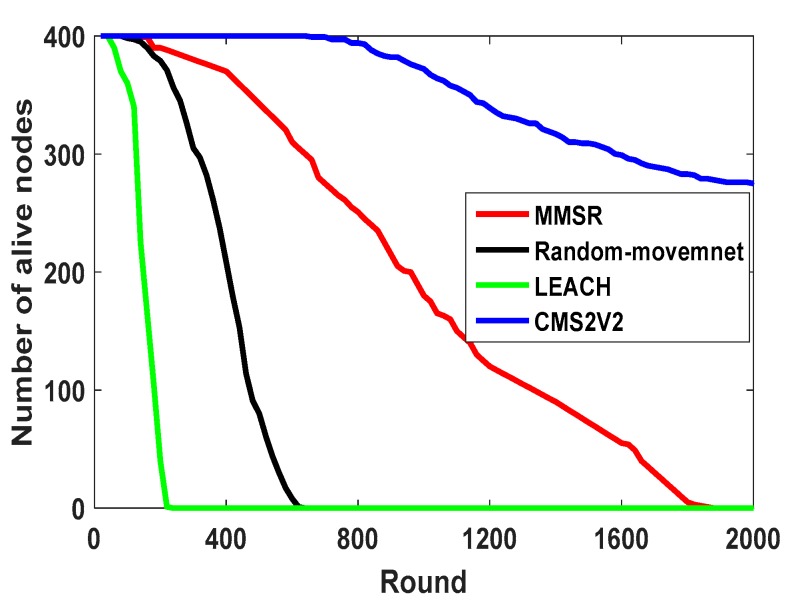
Number of alive nodes comparison.

From Figure 10, it can be seen that CM2V2 has better performance than the Random-Movement, MMSR and LEACH methods in terms of residual energy of the network. The residual energy of the LEACH and Random-Movement approach decrease more sharply than CM2V2 and MMSR, which means these two consume more energy than the CM2V2 technique during the routing process. Furthermore, full energy depletion of CM2V2 occurs later than with the three other methods, therefore, CM2V2 outperforms these strategies in this aspect. This stems from lower energy consumption of CMS2V2 in comparison with the other ones. Figure 11 shows the number of alive nodes as the number of rounds increases. As can be seen from Figure 11, MMSR doesn’t have any alive sensor nodes around 1800 rounds, while the number of alive nodes of CM2V2 is 300 nodes in 2000 rounds. Then, we can see that our CM2V2 technique has better performance and longer network lifetime in comparison with MMSR, Random-Movement and the algorithm using one static sink. This is because the energy consumption is completely balanced among CHs located in the same sector and different layers in CMS2V2 algorithm.

### 5.4. Influence of the Velocity of MS1

**Figure 12:**
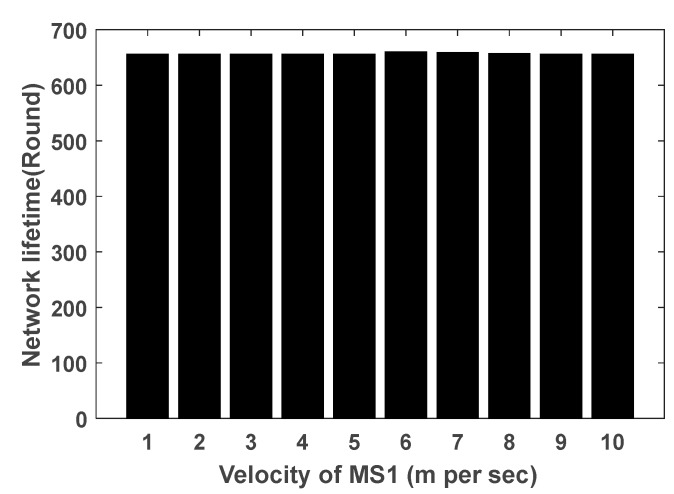
Network lifetime with different velocities of the innermost MS.

Figure 12 shows the efficacy of variation of the velocity of the innermost MS which has a constant velocity on network lifespan. Since the velocity of MSs are regulated based on the velocity of MS1, the network lifetime of the proposed method is evaluated by varying the velocity of MS1. As shown, this variation does not affect the network lifetime. Therefore, the CMS2V2 strategy can be used with different types of mobile agents with various velocities.

### 5.5. Balancing the Energy Consumption Ratio

**Figure 13:**
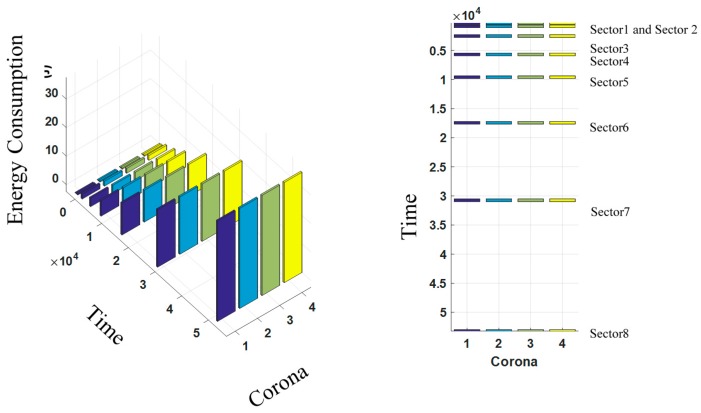
Energy consumption ratio of CHs.

Figure 13 shows the energy consumption ratio of CHs during different time periods in the CCWSN with four coronas and four sectors. In the CMS2V2 algorithm, MSs move along the predetermined paths based on the determined velocities and sojourn at the clusters for the limited time obtained from Equations (29) and (30). MSs compensate the extra energy consumption of CHs based on their waiting time calculated from Equation (24) to (27). As shown in Figure 13, the energy consumption ratio of CHs in the same sector and different coronas is balanced every time that MSs sojourn in the clusters in each sector. This feature is considered a solution to mitigate the energy holes, which increases the network lifetime.

## 6. Conclusions

In this paper, an optimal cluster size interval was calculated. The lower bound of this interval was calculated based on the density of nodes in the network. To determine the maximum value of interval and the exact value of the angle, a two-stage GA was proposed. Since one objective of employing clustering is to decrease the energy consumption of nodes, the power expenditure of sensors before applying clustering was also considered in our model. As a result, the energy consumption of MNs is remarkably reduced by using the method proposed in this work. We also proposed the CMS2V2 algorithm in order to mitigate the energy hole problem and extend the network lifespan. Finally, simulation results show that compared with other methods, our strategy can achieve better performance in terms of network lifespan. In addition, CMS2V2 could balance the energy consumption ratio of CHs in different time periods. In the future, we plan to determine the optimal sojourn time of MSs at different sites to solve the problem of the unbalanced energy consumption of sensor nodes.

## Figures and Tables

**Table 1:** Parameters Definition.

Parameter	Define the Parameters
n	Path loss exponent
α	Energy dissipated in the op-amp
r	Width of each corona
$E_{elec}$	The electrical energy consumption
$E_{tx}$	Energy usage for data transmission
$E_{rx}$	Energy consumption for receiving data
k	The number coronas
M	Total number of nodes
$N_{i}$	The number of nodes in *i*th corona
R	The network radius
$\varepsilon_{0}$	Initial energy of each corona
$E_{i}$	The energy expenditure *i*th corona
l	Packet Length

**Table 2:** Minimum and maximum number of sectors.

**Number of nodes (1st corona)**	6	12	18	25	31	37	43	50	56	62	68	75	81	87	93
**Number of sub-coronas**	1	2	2	2	2	3	3	3	3	3	4	4	4	4	4
**Number of sectors with minimum angle**	6	3	4	6	7	4	4	5	6	6	4	4	5	5	5
**Number of sectors with maximum angle**	4	3	3	3	3	3	3	3	3	3	3	3	3	3	3

**Table 3:** Parameters used in CM2V2.

Parameter	Value
K	2
SN	4
R	400 m^2^
D	200
Initial energy	0.2~2.1 J
Number of Nodes	400
transmitter amplifier	1e-11
Velocity of MS1	0.5 (m/s)
Packet Size	1000 bits
